# Potential role of vitamin D levels in amyotrophic lateral sclerosis cognitive impairment

**DOI:** 10.1007/s10072-023-06751-7

**Published:** 2023-03-23

**Authors:** Fabiola De Marchi, Massimo Saraceno, Maria Francesca Sarnelli, Eleonora Virgilio, Roberto Cantello, Letizia Mazzini

**Affiliations:** 1 ALS Centre, Neurology Unit, Department of Translational Medicine, Maggiore della Carità Hospital, University of Piemonte Orientale, Novara, Italy; 2 Neurology Unit, Department of Translational Medicine, Maggiore Della Carità Hospital, University of Piemonte Orientale, Novara, Italy

**Keywords:** Amyotrophic lateral sclerosis, Vitamin D, Cognitive impairment, Biomarkers, Prognosis

## Abstract

Cognitive impairment (CI) is common in amyotrophic lateral sclerosis (ALS): a keystone is identifying factors that could potentially modify the CI course. In recent years, vitamin D is becoming a potential modificatory factor for CI in many neurological disorders. This study aimed to highlight if vitamin D deficiency correlated with CI and clinical features in a cohort of ALS patients. We included 55 ALS patients with a neuropsychological evaluation (classified with the Strong Criteria) and a vitamin D dosage at the diagnosis. We also reviewed medical records and completed data for medical history, physical and neurological examination, and functional scales. At the diagnosis, 30 patients (54%) had CI. Most patients (82%) displayed low vitamin D levels (19.87 ± 9.80 ng/ml). Comparing the vitamin D level between patients with and without CI, we observed significantly lower values in the first group (15.8 ± 8.2 vs. 22.0 ± 9.7 ng/ml, *p*: 0.04). In the spinal female subgroup (*n* = 15), we found an inverse correlation between vitamin D and bizarreness score in the cognitive estimates test (*r* = 0.58; *p*: 0.04) and a positive correlation with the Corrected Raven’s Standard Progressive Matrices (*r* = 0.53, *p*: 0.04). Conversely, in the bulbar female group, we observed a correlation with the corrected direct span (*r* = 0.84, *p*: 0.03). With the log-rank survival analysis, we found that the patients with vitamin D < 10 ng/ml had a shorter disease duration (Chi: 5.78, *p*: 0.02). Our results indicate that levels of vitamin D can influence the cognitive status of people living with ALS and that severe deficits might be an adverse prognostic survival factor.

## Introduction

Amyotrophic lateral sclerosis (ALS) is a neurodegenerative disease involving the upper and lower motoneurons. It is the most frequent neuromuscular disease, with an incidence between 0.6 and 3.8 per 100,000 in Europe [1]. The median survival ranges from a few months to several years, generally between 3 and 5 years [2]. The clinical ALS phenotype could be divided into subtypes based on the onset of the symptoms, with different clinical patterns and prognoses [3]. Currently, the only approved drugs are riluzole and edaravone, with modest effects on the disease course; although, for some genetic forms, there are intriguing gene therapies on the horizon [4]. In the current absence of disease-modified treatments, the goal focuses on improving the quality of patients’ life by reducing and anticipating the problems that gradually arise, such as nutritional and pneumological [5].

Even if for years considered an exclusively motor disease, the role of cognitive impairment in ALS has overwhelmingly emerged in the last two decades. Cognitive disorders are present in up to 40% of patients, varying from dysexecutive or behavioral syndromes to frontotemporal dementia (10–15% of cases) [6, 7]. In the literature, cognitive impairment is associated with worse survival outcomes [8]. The Strong criteria [9] classified the different patterns of neuropsychological impairment as ALC-Ci (executive dysfunction), ALS-Bi (apathy with or without behavior modification), ALS-CiBi (cognitive and behavioral impairment), and ALS-FTD (association with frontotemporal dementia). At least partially, cognitive phenotypes correlated to clinical and demographic characteristics [3]; however, to date, no strong correlations are observed between fluid biomarkers and cognitive status in ALS [10].

Vitamin D is a calcium and phosphate metabolism molecule, activated at the dermal, renal, and hepatic levels to 1,25(OH)2D [11]. In the active form, they can activate strategic genes by binding specific intracellular receptors [12]. In humans, circulating 25(OH)D and metabolites do pass the blood-brain barrier by diffusion and enter neuronal and glial cells and are converted to 1,25(OH)2D [13], acting as a neurosteroid. Vitamin D affects neurons by reducing the inflammatory status, increasing neuronal growth factors, and favoring neuronal survival [14, 15]. The vitamin D deficiency is a global public health issue: low levels are also a risk factor in osteomalacia, cardiovascular diseases [16], diabetes [17], and psychiatric diseases [18]. In a large Italian Cohort, roughly 60% of females and 55% of men showed 25(OH)D deficient levels (< 20 ng/ml) [19].

In the literature, some studies investigated the role of vitamin D in ALS. ALS patients are reported having vitamin D levels slightly lower than the normal range [20, 21]. However, the results regarding the correlation between vitamin D and the diagnostic and prognostic role are inconclusive, actually making it impossible to consider vitamin D level as diagnostic and prognostic biomarker. In 2015, the group of Corcia reported a significantly higher level of bulbar than that in spinal-onset ALS patients and a negative association with body mass index [22]. Similarly, Paganoni et al. showed that 25(OH)D levels were negatively associated with baseline gross motor ALSFRS-R scores but did not predict the rate of disease progression [23]. Also regarding prognosis, studies reported conflicting results [22, 24, 25] without a certain trend. Moreover, limited results have been reported with the supplementation of Vitamin D in ALS patients, only in a trial with a high dose of vitamin D a slightly improvement of ALSFRS-R score were noted [26].

Interestingly, unlike what is reported in other neurodegenerative diseases [27–29], no prior study has tested whether vitamin D is involved in the development of cognitive impairment in ALS. This work aimed to identify a potential correlation between vitamin D and cognitive decline in ALS, and the eventual correlation with the clinical-demographic features and the potential prognostic effect.

## Materials and methods

### Study design and population

We conducted a retrospective study from January 2016 to January 2022, including patients evaluated at the Tertiary ALS Center “Maggiore della Carità” University Hospital, Novara, Italy.

We enrolled patients with the following criteria:

1. A probable supported by laboratory or definite ALS according to the El Escorial revised criteria [30, 31]
2. Age over 18 years old
3. A complete neuropsychological battery obtained within 3 months from the diagnosis
4. A vitamin D value obtained within three months from the diagnosis

In addition, we excluded patients with psychiatric/neurological diagnoses other than ALS or comorbidities which could affect vitamin D levels.

Of the included patients, we reviewed medical records and complete data on medical history, physical and neurological examination, the ALS Functional Rating Scale–Revised (ALSFRS-R) score [32–34], body mass index (BMI), and forced vital capacity percentage (FVC%) at baseline. We calculated the rate of progression from symptoms’ onset to vitamin D sample collection using the formula: (48-ALSFRS-R at sample collection)/(months from symptoms’ onset to sample collection). Subsequently, based on the median delta progression, we divided patients as “slow progressors” (≤ of median) or “fast progressors” (> of median). Survival time was calculated at the time of death (or tracheostomy); for alive patients were set the 15th October 2022 as censored data. We also collected the scores obtained with a complete neuropsychological test battery, and classified the cognitive status according to the Strong classification (ALS-normal, ALS-Bi, ALS-Ci, ALS-CiBi, ALS-FTD) [9].

### Neuropsychological evaluation

We used a neuropsychological battery to evaluate the global cognitive function and to operate a multi-domain evaluation. The following neuropsychological tests were administered to all patients by an ALS expert neuropsychologist: the short story test (prose memory) [35], the frontal assessment battery (global executive dysfunction) [36], the trial-making test (selective spatial attention and attentional shift) [37], the cognitive estimates (ability to provide quantity estimates, executive function) [38], the direct (verbal short-term memory) and inverse span (working memory, executive functions) [39], the clock drawing test (assesses long-term attention, memory, motor programming, executive function) [40], the Raven’s Standard Progressive Matrices (logical deductive reasoning) [41], and the verbal fluency (access to the phonological lexicon, linguistic domain) [42]. Where required, the tests were corrected for age and educational level. The evaluation lasted an average of forty minutes and was preceded by a premorbid psychological data evaluation.

### Vitamin D

Serum samples were measured with the LIAISON® 25OH VitD total assay certified since 2014 (DiaSorin Inc., 1951 Northwestern Ave—Stillwater, MN 55082—USA), an in vitro chemiluminescent immunoassay (CLIA). The resulting signal is directly proportional to the analyte concentration with a range of detection between 4.0 and 150 ng/ml. Board-certified laboratory technicians analyzed the samples. All measurements were performed according to the manufacturers’ instructions and in the same laboratory. For determining vitamin D deficiency or insufficiency, the following parameters set by the Italian endocrine society were used: > 30 optimal level, 21–30 ng/ml insufficiency, 10–20 ng/ml deficiency, < 10 severe deficiency [43].

### Statistical analysis

Statistical analyses were performed using IBM SPSS Statistics for Windows, version 27.0. (Armonk, NY: IBM Corp.) and Graphpad Prism 9 for MacOS (Graphpad Software, La Jolla, CA, USA). In order to make proper comparisons between different neuropsychological tests, we derived adjusted raw scores for age, sex, and education using the Italian normative scores. As required, the quantitative variables were synthesized using the following indices: mean, standard deviation (SD), median and interquartile range (IQR). Absolute and relative frequencies were used for qualitative variables. Preliminarily, we used the D’Agostino and Spearman normality test, providing good evidence that our data were normally distributed. Therefore, we used parametric/non-parametric tests to check differences between the various populations and the Pearson and Spearman coefficient for correlation among the variables studied, as required. As appropriate, correlations were corrected for age (by using the binomial logistic regression). Also, we evaluated the analysis both without adjustments for multiple testing and with a Bonferroni correction applied for three variables (sex, phenotype, rate of progression). All statistical testing requires a nominal *p* value of 0.01 to reach significance following Bonferroni correction (*p* = alpha/n, alpha = 0.05, *n* = 3).

We also use the Kaplan-Meier curves (Mantel-Cox test) and the Cox regression analysis to calculate survival curves. In all analyses, we considered *p* value < 0.05 as statistically significant.

The Hospital Maggiore della Carità’s research ethics committee reviewed and approved this study.

## Results

### Study population characteristics

A total of 55 patients were included in our analysis, of which 33 were male (60%). Forty-two patients (76%) had a spinal onset, and 13 had a bulbar onset (24%). The mean age at diagnosis was 63.69 ± 9.01 years. The mean ALSFRS-R at baseline was 39.74 ± 5.48, with a delta progression of 1.14/month (± 1.24). At the diagnosis, 30 patients (54%) had cognitive impairment: in detail, twenty-five patients were classified as ALS-Ci (45%), three as ALS-FTD (5%), and two ALS-Bi (4%). We did not observe cognitive impairment in 25 patients (46%, ALS-no). All patients’ features are summarized in Table 1.

**Table 1:** Clinicodemographic analysis of the included patients (*N* = 55)

Clinical features	Number = 55
Age at onset (mean ± SD)	63.69 ± 9.01
Male/Female (*N*)	33/22
Spinal / Bulbar onset (*N*, %)	42 (76%)/13 (24%)
ALSFRS-R score at diagnosis (mean ± SD)	39.7 ± 5.4
Monthly ΔALSFRS-R ± SD*	1.14 ± 1.24
BMI at diagnosis (mean ± SD)	25.6 ± 5.5
FVC% at diagnosis (mean ± SD)	82.1 ± 22.5
Cognitive status	
- Normal (*N*, %)	25 (46%)
- ALS-Ci/ALS-Bi/ALS-FTD (*N*, %)	25 (45%)/2 (4%)/3 (5%)

*Calculated in the period from disease onset to diagnosis

The table describes the clinicodemographic features of the enrolled patients at baseline, and the monthly progression is calculated from the disease onset to enrollment (*N* number, *SD* standard deviation, *ALSFRS*-*R* Amyotrophic Lateral Sclerosis Functional Rating Scale– Revised, *BMI* body mass index, *FVC*% force vital capacity %, *ALS*-*Ci* cognitive impairment, *ALS*-*Bi* behavioral impairment, *ALS*-*FTD* frontotemporal dementia)

### Vitamin D levels and clinicodemographic features

Most patients (82%) displayed low vitamin D levels, (mean of 19.87 ± 9.80 ng/ml in the whole sample): 9 (16%) patients had a severe deficiency (< 10 ng/ml), 23 (42%) a deficiency (10–20 ng/ml), 13 (24%) an insufficiency (20–30 ng/ml), and only 10 (18%) an optimal concentration (> 30 ng/ml). The level of vitamin D was similar between males and females (19.61 ± 9.64 and 20.26 ± 10.28 ng/ml, *p* value = 0.81). We did not observe any correlation with age (*r* = 0.06, *p* value: 0.68). Spinal-onset patients had lower level of vitamin D (18.72 ± 9.4 ng/ml) compared to bulbar-onset (23.59 ± 10.54; *p* value: 0.11).

Of our cohort, 14 patients were sampled in winter, eight in summer, 14 in spring, and 19 in autumn; no significant differences were observed in mean levels of vitamin D among the groups (data not shown).

Categorizing the levels of vitamin D into four groups (< 10, 10–20, 20–30, > 30 ng/ml), we did not observe any difference in age at onset, ALSFRS-R, rate of progression, FVC%, and BMI between the groups.

### Vitamin D and cognitive status

**Fig. 1:**
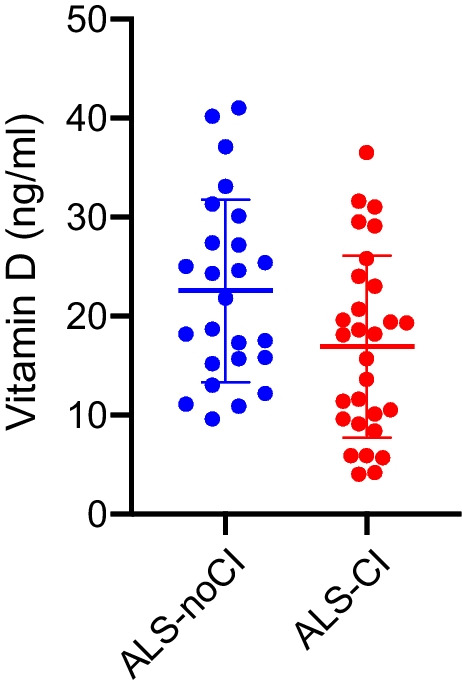
Comparison of vitamin D levels in ALS patients without cognitive impairment (ALS-noCI, blue) and with cognitive impairment (ALS-CI, considered as ALS-Ci + ALS-Bi + ALS-FTD, red)

Within the whole sample, comparing the vitamin D level between patients with and without cognitive impairment, we observed significantly lower values in the first group (15.8 ± 8.2 ng/ml) versus the second one (22.0 ± 9.7 ng/ml, *p* value: 0.04, corrected for age) (Fig. 1).

Then, to evaluate in which subgroups of patients the level of vitamin D was more influential on cognitive deficits, we compared patients based on phenotype, sex, and rate of disease progression.

**Fig. 2:**
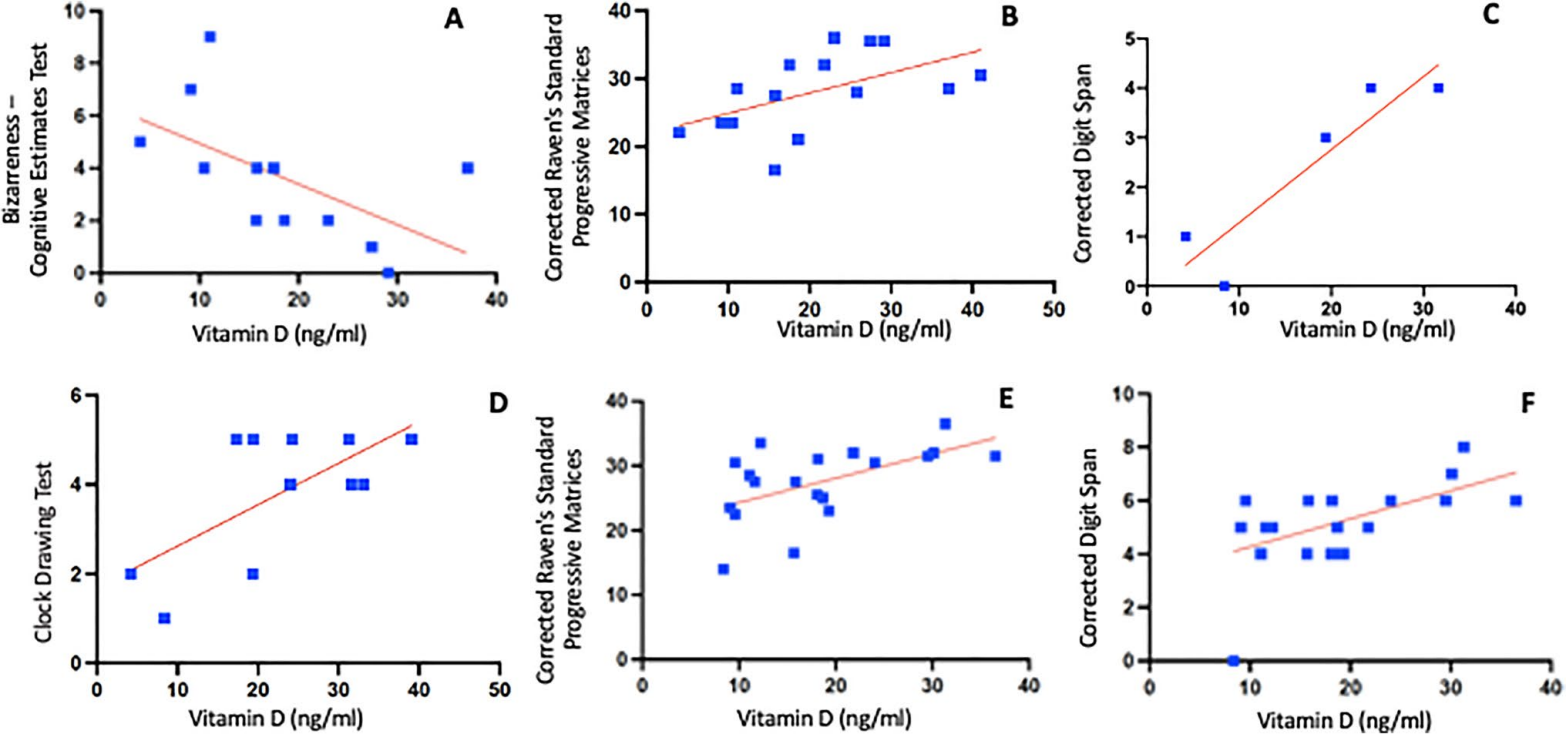
Significant correlation between vitamin D level and neuropsychological scores. From panel **A** and panel **B**: correlations between the vitamin D level in female spinal patients and the neuropsychological tests; panel **C**: correlations between the vitamin D level in female bulbar patients and the neuropsychological tests; panel **D**: correlations between the vitamin D level in bulbar patients and the neuropsychological tests; from panel **D** to panel **E**: correlations between the vitamin D level in fast progressor patients and the neuropsychological tests. X-axis: vitamin D values; Y-axis: neuropsychological scores. **F**: **E**: correlations between the vitamin D level in fast progressor patients and the neuropsychological tests. X-axis: vitamin D values; Y-axis: neuropsychological scores

In the spinal female subgroup (*n* = 15), we found an inverse correlation between vitamin D and bizarreness score in the cognitive estimates test (*r* = 0.58; *p* value: 0.04; panel A) (Fig. 2) and a positive correlation between vitamin D plasma level and score obtained to Corrected Raven’s Standard Progressive Matrices (*r* = 0.53, *p* value: 0.04, panel B) (Fig. 2). Conversely, in the bulbar female group, we observed a direct correlation between vitamin D level and the corrected direct span (*r* = 0.84, *p* value: 0.03, panel C) (Fig. 2). In the whole group of bulbar patients (*n* = 13), a significant positive correlation emerged between vitamin D level and the clock drawing test (*r* = 0.45, *p* value: 0.02, panel D) (Fig. 2) and a positive trend with the corrected direct span (*r* = 0.32, *p* value: 0.07).

Regarding the disease progression, in patients classified as fast progressors (*n* = 18), we observed a moderate positive correlation between the Corrected Raven’s Standard Progressive Matrices (*r* = 0.31, *p* value: 0.01; panel E) (Fig. 2) and the corrected direct span (*r* = 0.32; *p* value: 0.01, panel F) (Fig. 2), and vitamin D level; meaning that higher values of vitamin D are associated with better neuropsychological scores. After correction for multiple comparisons with the Bonferroni method, only correlations with the rate of progression have remained significant.

Lastly, analyzing the significant tests in the four groups (< 10, 10–20, 20–30, > 30 ng/ml), we confirmed that patients with vitamin D < 10 ng/ml had significantly lower scores in the Corrected Raven’s Standard Progressive Matrices (*p* value: 0.01).

### Vitamin D and survival

**Fig. 3:**
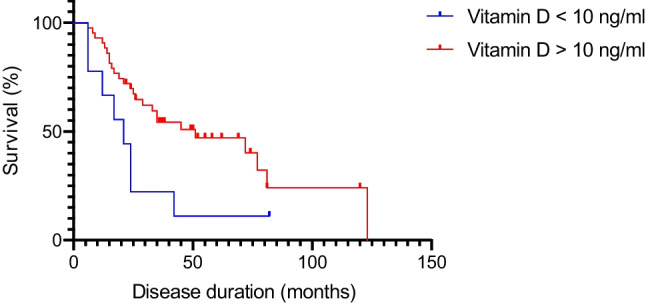
Survival analysis comparing disease duration in patients with vitamin D level < 10 ng/ml (blue line) and > 10 ng/ml (red line). In Cox regression model, Chi: 7.78 and *p* value: 0.02. X-axis: disease duration in months (for alive patients, we considered the 15th October 2022 as censored data; y-axis: percentage of survival over time

The median survival time of the whole cohort was 31 months (IQR: 17–51.25). With the Cox regression survival analysis (two variables, vitamin D levels and age), we found that the patients with a severe deficit of vitamin D (< 10 ng/ml) had a shorter disease duration compared to the other participants (median survival: 21.00 vs. 51.00 months; global Chi: 7.78, *p* value: 0.02, Fig. 3). In a univariate model, no other factor was singularly significantly associated with a shorter disease duration (data not shown). However, in a Cox multivariate analysis including age at diagnosis, cognitive status, diagnostic delay, phenotype, sex, ALSFRS-R, and BMI at baseline, vitamin D levels were not independently significant, even maintaining a positive trend (Chi: 3.29, SE: 0.496, *p* value: 0.07).

## Discussion

This study, for the first time, revealed a correlation between the plasmatic values of vitamin D and cognitive impairment in ALS, especially concerning executive and reasoning functions. In this regard, firstly, we observed lower vitamin D levels in patients with cognitive impairment than those without and correlations with cognitive tests and vitamin D levels. Additionally, as clinical correlations, we observe a possible negative influence of vitamin D deficiency on the disease duration. On the contrary, we did not observe significant correlations between the vitamin D levels and the main clinical features of our ALS cohort (e.g., sex, phenotype, age at onset, and age at enrollment).

A correlation between vitamin D levels, neurodegenerative diseases, and cognitive status is well-known in recent literature. For example, vitamin D levels are reported to be lower than expected values in Alzheimer’s disease and other dementias [44, 45] and in Parkinson’s disease (PD) [46, 47], where lower levels of vitamin D can represent a biomarker of risk of mild cognitive impairment (MCI) development in PD patients [48]. Furthermore, improvements in cognitive functions have been reported after supplementation in patients with MCI and Alzheimer’s disease [49, 50].

Regarding motoneuron diseases, the role of vitamin D has yet to be studied thoroughly, and the available studies have provided contrasting results [20]. Also, the potential correlation between cognitive impairment and vitamin D has never been investigated. Previous studies have reported hypovitaminosis D in these patients [20, 22, 26], and possible clinical correlations with vitamin D levels. For example, Paganoni et al. showed direct correlations between vitamin D values and the motor subscores of the ALSFRS-R [23]. On the other hand, the group of Camu emphasized a possible inverse correlation between the levels of vitamin D and the functional status [51]. Our cohort reported a lower concentration in spinal compared to bulbar patients, even without statistical significance. These results may be related to a direct neuro (and muscular) protective action of the micronutrient, or only an indirect consequence of ALS, occurring through loss of mobility and insufficient exposure to sunlight for adequate synthesis. Always considering our cohort, no other relevant clinical correlations were observed.

Our most exciting results concern the correlation between hypovitaminosis D and cognitive impairment, assessed with a large battery of neuropsychological tests. Our assumption that vitamin D may play a role in cognitive impairment in ALS is derived from epidemiological and clinical findings in other neurodegenerative diseases, and our results support this hypothesis also in the ALS.

Dividing patients based on onset phenotype (i.e., spinal and bulbar), we observed two different patterns of hypovitaminosis D. In female spinal patients, we found an inverse correlation between vitamin D and bizarreness score in the Cognitive Estimates Test and a positive correlation between vitamin D plasma level and score obtained to Corrected Raven’s Standard Progressive Matrices. The first test is generally considered to be a measure of executive functions, while the second one is commonly used to obtain a non-verbal reasoning score, and measuring abstract reasoning. Instead in the whole group of bulbar patients, both males and females, a correlation emerged with the Clock Drawing Test and the Corrected Direct Span. These two tests are related to global cognition and memory, pointing out a possible correlation between low vitamin D values and worse overall performance perhaps related to more compromised patients globally [52]. The results of the Corrected Raven’s Standard Progressive Matrices test were also confirmed in patients classified as rapidly progressing and in patients with a severe deficit of vitamin D (< 10 ng/ml), thus resulting in the test being most correlated to hypovitaminosis D. The apparent predominance of some cognitive functions in a subgroup of female patients could be attributable to gender-related neurobiological characteristics, including the different hormonal structure and polymorphic variants in the vitamin D receptor [53, 54], that negatively influence the neurotrophic factors activity.

Also, based on the above-described analyses, we are inclined to consider hypovitaminosis D as one of the potential causes of cognitive impairment. However, the results could still be considered inversely, with the deficit of vitamin D secondary to cognitive impairment and more disabling disease. In fact, patients with a more severe disease phenotype (also due to the concomitant cognitive disorders) can have more difficulty eating and walking out from home, and faster catabolism, with hypovitaminosis D could be a subsequent phenomenon.

Finally, the survival analysis revealed a deleterious effect of severely low levels of vitamin D concentrations on the disease duration, independently of functional status at baseline and BMI, suggesting that this relation may be direct and independent of the nutritional status. This finding has had contrasting results [22, 24], so interpreting this result should be prudent and must be replicated in more extensive studies. However, it remains an exciting result, highlighting a possible negative modifiable survival factor. In support of the robustness of our data, an elegant recent study published by Sutherland and colleagues, analyzing the mortality in a large group of subjects from the UK Biobank, observed that the risk for death decreased with increasing vitamin D concentrations [55].

Strengths of this study include the novelty of association in ALS, the accurate clinical characterization of patients, and their monitoring throughout the disease course. However, this study is not without limitations. First, the sample size is limited, so we had to group patients with different cognitive impairments (ALS-ci, ALS-bi, ALS-FTD), losing other differences between groups. Second, we did not include variables that could have influenced the vitamin D data acting as modifying factors, such as smoking, physical activities, and other blood chemistry dysfunctions. Also, we did not evaluate the eventual co-presence of depression, a factor that can negatively influence neuropsychological tests. Lastly, some patients did not perform the vitamin D blood test and neuropsychological evaluation on the same day and, taking into account the rapid metabolism and variability, there may be theoretical discrepancies in the vitamin D values between the day of the sample and the day of the neurocognitive tests.

## Conclusion

Our results indicate that levels of Vitamin D can influence the cognitive status of people living with ALS, and that severe deficits might be an adverse prognostic survival factor. From a gender medicine perspective, fascinating is the observation that some neuropsychological tests were significantly more altered in females than males: this finding should be better investigated, as well as evaluating gender-related molecular and pathophysiological differences. Lastly, our data will be helpful as a starting point to focus on this micronutrient during the diagnostic phase, with the idea that a possible correction can act as a modifying neuroprotective factor.
